# Association of serum uric acid to high-density lipoprotein cholesterol ratio with all-cause and cardiovascular mortality in patients with diabetes or prediabetes: a prospective cohort study

**DOI:** 10.3389/fendo.2024.1476336

**Published:** 2024-12-05

**Authors:** Xiaoli Lai, Tao Chen

**Affiliations:** ^1^ Department of Endocrinology and Metabolism, Longyan First Affiliated Hospital of Fujian Medical University, Longyan, China; ^2^ The Third Clinical Medical College, Fujian Medical University, Fuzhou, China

**Keywords:** UHR, mortality, diabetes, prediabetes, NHANES, J-shaped association

## Abstract

**Background and aims:**

The serum uric acid (UA) to high-density lipoprotein cholesterol (HDL-C) ratio (UHR) is a novel biomarker that indicates inflammation and metabolic disorders. Also, it has been shown that UHR correlates with the risk of cardiovascular disease. Despite this, limited research exists on its prognostic significance. This study aimed to explore the association of UHR with all-cause and cardiovascular mortality in patients with diabetes or prediabetes.

**Methods:**

This cohort study included 18,804 participants from the National Health and Nutrition Examination Survey (NHANES) 2005-2018 with diabetes or prediabetes aged 20 years or older, followed until December 31, 2019. Patients with diabetes or prediabetes were grouped according to quartiles of UHR, which was calculated as serum UA (mg/dL)/HDL-C (mg/dL). Kaplan-Meier survival analysis, multivariable Cox proportional hazards regression models, restricted cubic spline analysis, and threshold effects were performed to assess the association between baseline UHR and all-cause and cardiovascular mortality. Subgroup analysis and sensitivity analysis were also conducted.

**Results:**

During a median follow-up of 80 months, a total of 2,748 (14.61%) deaths occurred, including 869 (4.63%) cardiovascular deaths. Kaplan-Meier survival analysis revealed that the highest quartile of UHR had the highest mortality rates. Multivariable Cox regression analysis indicated that individuals in the highest quartile of UHR had a significantly higher risk of all-cause mortality (HR: 1.24, 95% CI: 1.07-1.45) and cardiovascular mortality (HR: 1.56, 95% CI: 1.19-2.04) compared to those in the second quartile. A J-shaped association between UHR and both all-cause and cardiovascular mortality was observed, with threshold points of 13.73% and 9.39%, respectively. Specifically, when UHR was above the respective thresholds, the HRs of a 10% increment of UHR for all-cause mortality and cardiovascular mortality were 1.45 (95% CI: 1.31-1.61) and 1.38 (95% CI: 1.20-1.60). However, UHR below the threshold did not significantly correlate with mortality. Furthermore, subgroup analyses showed that the correlation of UHR with all-cause mortality was significantly modified by sex and age, with a persistent positive correlation observed in women and those aged < 60.

**Conclusion:**

Higher UHR was correlated with increased all-cause and cardiovascular mortality in patients with diabetes or prediabetes.

## 1 Introduction

The prevalence of glucose metabolism disorders is increasing globally due to shifts in lifestyle and population aging, presenting a significant challenge to public health. According to the International Diabetes Federation (IDF), approximately 720 million people worldwide were prediabetic, and 537 million were diabetic in 2021, which is expected to reach 1 billion and 783 million people by 2045, respectively (1). Prediabetes, characterized as an intermediate state between normoglycemia and diabetes, has been proven to be correlated with the onset of diabetes and elevated risks of cardiovascular disease, chronic kidney disease, cancer, and other diseases, all of which pose a considerable health burden (2–7). Furthermore, cumulative evidence has indicated that both diabetes and prediabetes increase the risk of all-cause and cardiovascular death (3, 8). Thus, earlier identification and intervention of risk factors are essential to improve the prognosis of individuals with abnormal glucose metabolism.

Serum uric acid (UA) to high-density lipoprotein cholesterol (HDL-C) ratio (UHR), a novel inflammation and metabolic indicator, has gained attention in recent years (9). Zhou et al. (10, 11) reported that UHR is an effective marker for predicting insulin resistance (IR), irrespective of the presence of type 2 diabetes mellitus (T2DM). Several other studies have shown that elevated UHR is linked to metabolic diseases such as metabolic syndrome, non-alcoholic fatty liver disease, and prediabetes (12–17). Additionally, elevated UHR has been found to be associated with coronary artery disease, ischemic cardiomyopathy, and arterial stiffness (18–21). UHR could also predict adverse cardiovascular outcomes and all-cause mortality risk in patients with acute myocardial infarction (22). Furthermore, a high cumulative UHR has been connected with the incidence and progression of chronic kidney disease (23). Increasing evidence has suggested that UHR, as a simple and easily obtainable composite indicator, may hold predictive and prognostic value in clinical settings.

Abnormal glucose metabolism can often coexist with other metabolic disorders, such as UA metabolism disorders and dyslipidemia (24, 25). These interconnected metabolic disorders can mutually influence each other, increasing the risk of cardiovascular diseases and negatively impacting prognosis. Although previous studies have confirmed the predictive capabilities of UHR for metabolic diseases and its correlation with cardiovascular diseases, limited research has delved into the association of UHR with mortality risk. Therefore, this study aimed to explore the association of UHR with all-cause mortality and cardiovascular mortality in patients with diabetes or prediabetes.

## 2 Materials and methods

### 2.1 Study design and participants

This prospective cohort study utilized data from the National Health and Nutrition Examination Survey (NHANES), a research program funded by the Centers for Disease Control and Prevention, to evaluate Americans’ health and nutritional status. The survey employs a nationally representative sample design involving complex stratified, multistage, probability sampling and is carried out biennially. The studies involving human participants received approval from the Research Ethics Review Board of the National Center for Health Statistics (NCHS), and all participants provided written informed consent. Relevant data can be accessed at https://www.cdc.gov/nchs/nhanes/index.htm.

**Figure 1:**
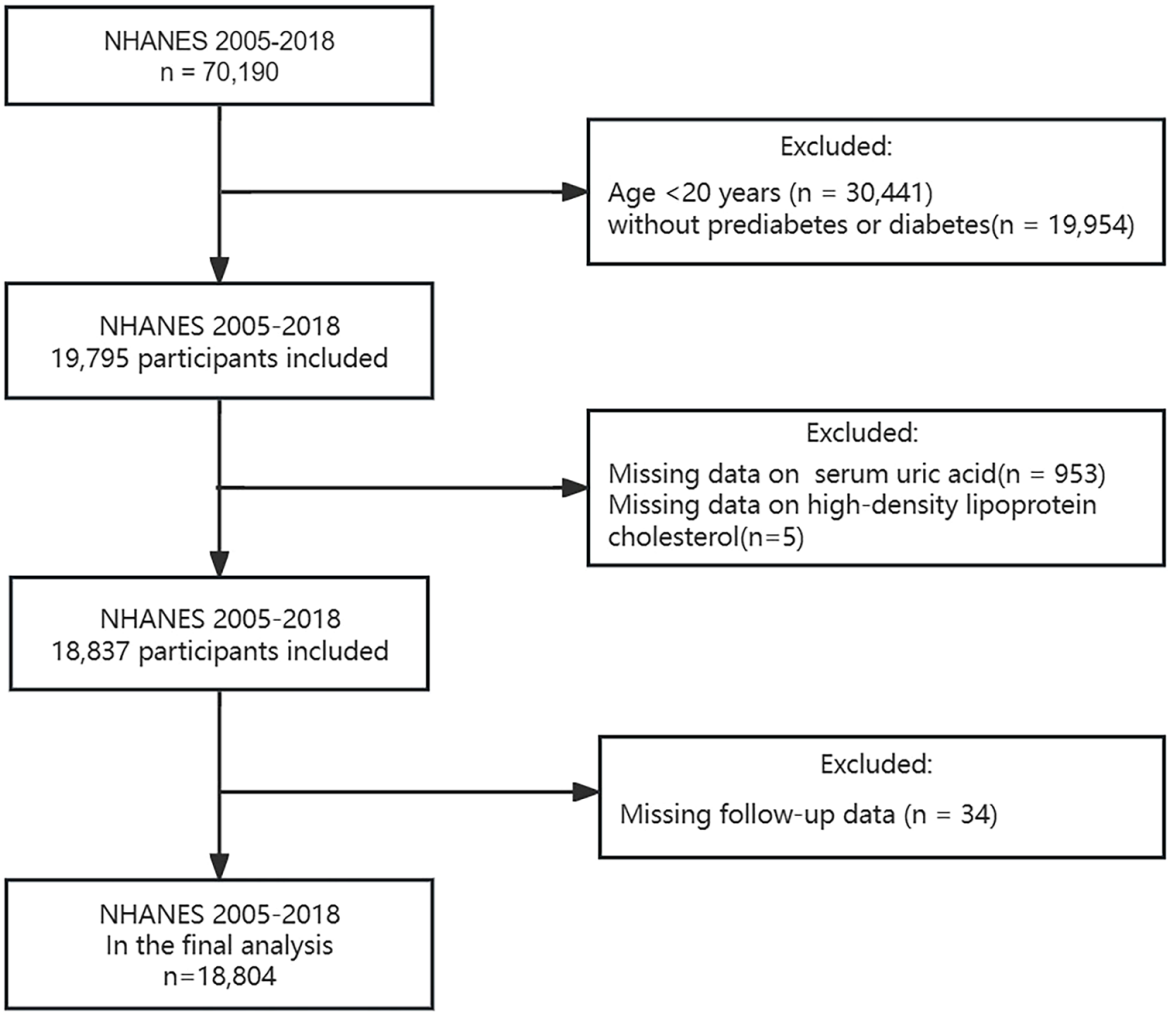
Flowchart of study participants selection.

According to the 2024 diagnostic criteria from the American Diabetes Association (ADA) guideline (26), diabetes was defined as: (1) a fasting plasma glucose level ≥ 7.0 mmol/L; (2) a random plasma glucose level or 2-h post-load plasma glucose (75g oral glucose tolerance test) ≥ 11.1 mmol/L; (3) a glycosylated hemoglobin A1c (HbA1c) level ≥ 6.5%; (4) self-reported diabetes diagnosis; (5) use of antidiabetic medication. Prediabetes was defined as: (1) a fasting plasma glucose level of 5.6-6.9 mmol/L; (2) a random plasma glucose level or 2-h post-load plasma glucose (75g oral glucose tolerance test) of 7.8-11.0 mmol/L; (3) an HbA1c level of 5.7-6.4%; (4) self-reported diagnosis. A total of 70,190 individuals were reviewed during NHANES 2005-2018. Based on the diagnostic criteria above, 19,795 adults aged 20 years and older with diabetes or prediabetes were initially screened. After excluding participants lacking serum UA (n = 953), HDL-C (n = 5), and follow-up data (n = 34), 18,804 participants were ultimately included (**Figure 1**).

### 2.2 Calculation of UHR

UHR was calculated as the ratio of serum UA (mg/dL) to HDL-C (mg/dL). Serum UA levels were determined using the timed endpoint method, while HDL-C levels were determined using the direct immunoassay method. For detailed guidelines on laboratory storage, measurement procedures, and quality control, please refer to the NHANES website. Participants were divided into four categories (Q1, Q2, Q3, and Q4) by quartile of UHR.

### 2.3 Outcome ascertainment

Mortality data was obtained from National Death Index (NDI) death certificate records provided by NCHS, and the International Statistical Classification of Diseases, 10th Revision (ICD-10) was used to identify death causes. Follow-up time was determined between the date of the baseline examination and death or December 31, 2019, whichever occurred first. The study outcomes were all-cause mortality and cardiovascular mortality. Cardiovascular deaths included death from heart disease (codes I00–I09, I11, I13, I20–I51) and cerebrovascular disease (codes I60-I69). For more detailed information on mortality data, please visit https://www.cdc.gov/nchs/data-linkage/mortality.htm.

### 2.4 Covariates

Based on prior experience, several potential confounding covariates were included in the present study. Demographic information included sex (male, female), age, race (non-Hispanic white, non-Hispanic black, other Hispanic, Mexican American, and others), educational level (less than high school, high school or GED, college or above), marital status (married/living with partner, living alone), family income-poverty ratio (PIR) (< 1.3, 1.3-3, ≥ 3) and body mass index (BMI) (< 18.5 kg/m^2^, 18.5-25 kg/m^2^, 25-30 kg/m^2^, ≥ 30 kg/m^2^). Lifestyle included smoking status (never, former, current) and alcohol intake (mild, moderate, heavy). Specifically, individuals who consumed three or more drinks per day for women and four or more drinks per day for men, with five or more binge drinking days per month, were classified as heavy alcohol drinkers; those who consumed two drinks per day for women and three drinks per day for men, with two binge drinking days per month, were classified as moderate alcohol drinkers. Those who did not meet the above criteria were considered mild alcohol drinkers. Comorbidities included cardiovascular disease, chronic kidney disease, hypertension, hyperlipidemia, and cancer. Cardiovascular disease refers to any one or combination of self-reported coronary heart disease, congestive heart failure, heart attack, angina, and stroke. Medication use included self-reported use of antidiabetic drugs, lipid-lowering drugs, and uric acid-lowering drugs in the past 30 days. In addition, HbA1c was also collected. Detailed information on covariates can be found on the NHANES website.

### 2.5 Statistical analysis

Exam sample weights were used in the current study as recommended by the NHANES guidelines. Baseline characteristics were described according to UHR quartiles. Continuous variables were expressed as mean ± standard error (SE), and differences between groups were compared using weighted one-way analysis of variance (ANOVA). Categorical variables were expressed as weighted percentages (%), and differences between groups were compared using weighted chi-square tests. The proportional hazards assumption was verified by examining Schoenfeld residuals, and there were no violations of this assumption. Variance inflation factor (VIF) was used to quantify multicollinearity between the adjusted covariates, and all VIFs were < 5. All-cause and cardiovascular deaths were assessed separately for subjects in the UHR quartiles using a weighted Kaplan-Meyer survival analysis. Risk tables were utilized to provide the probability of survival at different follow-up durations (at 50-month intervals). Three weighted Cox proportional risk models were conducted to calculate hazard ratios (HRs) and 95% confidence intervals (95% CI) for the relationship of UHR quartiles with all-cause and cardiovascular mortality. The median value of each UHR category was used as a continuous variable in the models, and linear trend tests were performed. Model 1 was unadjusted, while Model 2 was adjusted for age and sex. Model 3 was further adjusted for race, educational level, marital status, PIR, BMI, smoking status, alcohol intake, cardiovascular disease, chronic kidney disease, hypertension, hyperlipidemia, cancer, and HbA1c based on Model 2. A multiple imputation was used for missing covariates. The dose-response relationship of UHR with all-cause and cardiovascular mortality was additionally examined using restricted cubic spline (RCS) analysis. Node selection for the RCS curve was guided by minimizing the Akaike information criterion (AIC). In the case of a non-linear relationship, the two-piecewise Cox proportional hazards regression analyses were employed to estimate thresholds through threshold effects. Subgroup analyses were conducted based on age (< 60 years, ≥ 60 years), sex (male, female), BMI (< 18.5 kg/m^2^, 18.5-25 kg/m^2^, 25-30 kg/m^2^, ≥ 30 kg/m^2^), cardiovascular disease (yes, no), chronic kidney disease (yes, no), hypertension (yes, no), hyperlipidemia (yes, no), and diabetic condition (prediabetes, diabetes). Interaction terms were used among the subgroups to assess potential effect modification, followed by a likelihood ratio test. Finally, two sensitivity analyses were performed: (1) Participants who died within two years were excluded to reduce potential reverse causality bias; (2) To mitigate the confounding effects of medications, additional adjustments for glucose-lowering medications (yes, no), lipid-lowering medications (yes, no), and uric acid-lowering medications (yes, no) were made based on model 3. All analyses were performed using R (version 4.3.3) and EmpowerStats (version 4.2.0, www.empowerstats.com; X&Y Solutions, Inc. Boston MA). *P* < 0.05 was considered statistically significant.

## 3 Results

### 3.1 Baseline characteristics of participants

A total of 18,804 participants (weighted number 98,575,031) were eligible for the study, including 12,071 prediabetics and 6,733 diabetics with a mean age (± SE) of 54.27 ± 0.22 years. Among all the participants, 48.55% were females, and 51.45% were males. The mean (± SE) of serum UA was 5.68 ± 0.02 mg/dL, and the mean (± SE) of HDL-C was 51.01 ± 0.23 mg/dL. The mean (± SE) of UHR of the enrolled patients was 12.31 ± 0.07%, and the participants were classified according to the UHR quartiles: 0.67% ≤ Q1 ≤ 8.50%, 8.50% < Q2 ≤ 11.40%, 11.40% < Q3 ≤ 15.20%, and 15.20% < Q4 ≤ 80.00%. Weighted baseline characteristics are listed in **Table 1**. Compared to those with the lowest UHR quartile (Q1), those with the highest (Q4) were more likely to be younger, male, Non-Hispanic White, less educated, married/living with a partner, economically deprived, obese, former or current smokers, and mild or heavy alcohol drinkers. They also had higher serum UA and HbA1c levels and lower HDL-C levels. Notably, as the UHR quartiles (from Q1 to Q4) increased, the proportion of participants with comorbid cardiovascular disease (from 10.80% to 18.65%), chronic kidney disease (from 17.18% to 27.88%), hypertension (from 45.38% to 60.05%), and hyperlipidemia (from 72.30% to 92.90%) increased significantly. All variables, except for history of cancer, exhibited statistically significant variations across the four groups (all *P* < 0.05).

**Table 1:** Characteristics of participants according to UHR quartiles.

Characteristic	All	UHR (%) quartiles								*P* value	
		Q1 [0.67, 8.50]		Q2 (8.50, 11.40]		Q3 (11.40, 15.20]		Q4 (15.20, 80.00]			
N of participants	18804	4728		4658		4667		4751			
UHR, %	12.31 ± 0.07	6.55 ± 0.03		9.96 ± 0.02		13.17 ± 0.02		19.45 ± 0.08		**< 0.0001**	
UA, mg/dL	5.68 ± 0.02	4.37 ± 0.02		5.27 ± 0.02		6.00 ± 0.02		7.05 ± 0.02		**< 0.0001**	
HDL-C, mg/dL	51.01 ± 0.23	68.30 ± 0.40		53.14 ± 0.18		45.70 ± 0.16		37.09 ± 0.15		**< 0.0001**	
HbA1c, %	6.08 ± 0.01	5.96 ± 0.02		6.05 ± 0.02		6.11 ± 0.02		6.18 ± 0.03		**< 0.0001**	
Age, years	54.27 ± 0.22	56.13 ± 0.33		54.67 ± 0.35		53.80 ± 0.33		52.50 ± 0.35		**< 0.0001**	
Sex (%)										**< 0.0001**	
Female	48.55	77.51		55.60		38.40		23.15			
Male	51.45	22.49		44.40		61.60		76.85			
Race (%)										**< 0.0001**	
Non-Hispanic Black	12.47	14.25		13.28		11.43		10.96			
Non-Hispanic White	64.50	63.78		62.14		66.13		65.82			
Other Hispanic	5.55	5.52		5.71		5.28		5.70			
Mexican American	9.07	8.51		9.59		9.24		8.97			
Others	8.42	7.94		9.27		7.92		8.55			
Education level (%)										**0.003**	
Less than high school	19.52	17.76		20.14		19.29		20.99			
High school or GED	24.78	23.46		25.44		24.42		25.91			
College or above	55.60	58.78		54.42		56.29		53.10			
Marital status (%)										**< 0.001**	
Married/living with a partner	65.18	61.83		64.61		66.69		67.70			
Living alone	34.76	38.17		35.39		33.31		32.30			
PIR (%)										**0.007**	
< 1.3	20.37	19.19		20.28		20.19		21.81			
1.3-3	28.38	26.51		27.97		28.49		30.50			
≥ 3	43.62	45.63		44.63		43.57		40.70			
BMI, kg/m^2^ (%)										**< 0.0001**	
< 18.5	0.92	2.63		0.78		0.15		0.13			
18.5-25	17.87	33.36		19.71		12.63		5.94			
25-30	32.12	34.90		33.01		32.44		28.18			
≥ 30	47.84	28.11		45.66		53.52		63.86			
Smoking status (%)										**< 0.0001**	
Never	51.30	56.38		53.59		50.31		45.16			
Former	29.40	25.73		26.98		30.72		34.11			
Current	19.24	17.89		19.43		18.97		20.73			
Alcohol intake (%)										**< 0.0001**	
Mild	61.18	58.39		62.56		61.39		62.41			
Moderate	13.05	17.42		13.30		11.61		9.89			
Heavy	15.27	12.79		13.36		16.93		17.90			
Cardiovascular disease (%)	14.42	10.80		12.41		15.73		18.65		**< 0.0001**	
Chronic kidney disease (%)	21.21	17.18		19.16		21.31		27.88		**< 0.0001**	
Hypertension (%)	52.60	45.38		50.63		54.21		60.05		**< 0.0001**	
Hyperlipidemia (%)	81.62	72.30		77.00		84.05		92.90		**< 0.0001**	
Cancer (%)	12.86	13.53		12.34		13.04		12.56		0.597	

Mean ± SE for continuous variables: P value for weighted one-way analysis of variance (ANOVA). % for categorical variables: P value for the weighted chi-square tests. UHR, uric acid to high-density lipoprotein cholesterol ratio; UA, uric acid; HDL-C, high-density lipoprotein cholesterol; PIR, family income-poverty ratio; BMI, body mass index. Significance differences were showed as P < 0.05 marked in bold.

### 3.2 Association of UHR with all-cause and cardiovascular mortality

**Figure 2:**
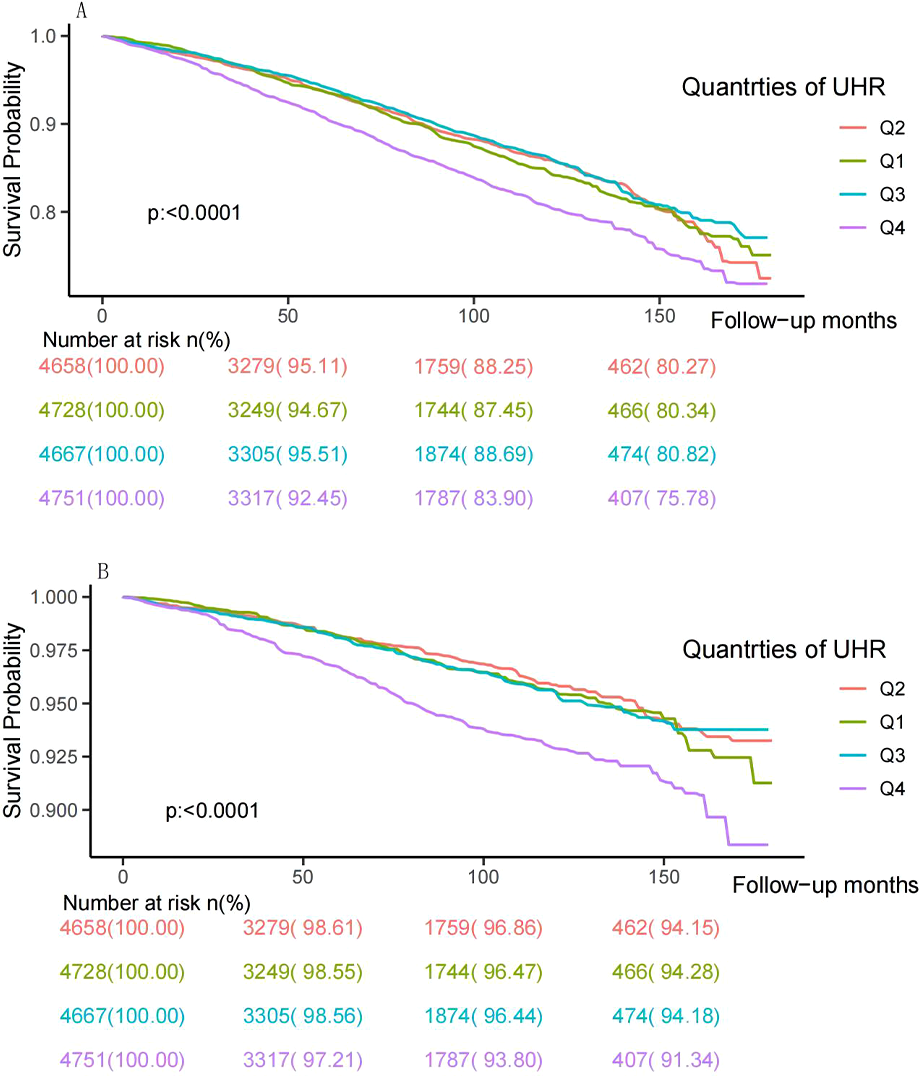
Kaplan–Meier survival curve and risk table for all-cause **(A)** and cardiovascular mortality **(B)** by UHR quartiles. UHR: uric acid to high-density lipoprotein cholesterol ratio.

During a median follow-up time of 80 months, 2748 (14.61%) deaths occurred, including 869 (4.62%) cardiovascular deaths. The Kaplan-Meyer survival analysis is displayed in **Figure 2**. A significant reduction in individual survival was observed when UHR levels were between 15.20% and 80% (Q4) (*P* both < 0.0001). Three Cox regression models were constructed to analyze the association of UHR qualities with mortality, and the second quartile (Q2) was set as the reference group, as detailed in **Table 2**. The unadjusted model showed that individuals in the highest quartile of UHR (Q4) had higher all-cause mortality (HR: 1.34, 95% CI: 1.17-1.53, *P* < 0.0001) and cardiovascular mortality (HR: 1.78, 95% CI: 1.41-2.24, *P* < 0.0001) compared to those in the second quartile (Q2). After fully adjusting for confounders, participants in group Q4 exhibited a 24% increased risk of all-cause mortality (HR: 1.24, 95% CI: 1.07-1.45, *P* = 0.005) and a 56% increased risk of cardiovascular mortality (HR: 1.56, 95% CI: 1.19-2.04, *P* = 0.001) compared to those in the Q2 group.

**Table 2:** Association between UHR and risks of mortality in patients with diabetes or prediabetes.

UHR quantiles		Model 1		Model 2		Model 3	
	Number of deaths	HR (95% CI)	*P* value	HR (95% CI)	*P* value	HR (95% CI)	*P* value
All-cause mortality							
**Q1**	620	1.02 (0.90, 1.16)	0.779	0.95 (0.83, 1.09)	0.446	1.01 (0.88, 1.16)	0.875
**Q2**	621	Reference		Reference		Reference	
**Q3**	653	0.94 (0.83, 1.07)	0.366	0.97 (0.85, 1.10)	0.614	0.91 (0.78, 1.05)	0.185
**Q4**	854	1.34 (1.17, 1.53)	**< 0.0001**	1.48 (1.28, 1.71)	**< 0.0001**	1.24 (1.07, 1.45)	**0.005**
***P* for trend**			**< 0.0001**		**< 0.0001**		**0.004**
Cardiovascular mortality							
**Q1**	181	1.07 (0.86, 1.35)	0.534	0.98 (0.78, 1.24)	0.880	1.16 (0.89, 1.50)	0.270
**Q2**	172	Reference		Reference		Reference	
**Q3**	216	1.07 (0.84, 1.37)	0.564	1.11 (0.86, 1.44)	0.431	0.99 (0.75, 1.30)	0.936
**Q4**	300	1.78 (1.41, 2.24)	**< 0.0001**	1.99 (1.54, 2.57)	**< 0.0001**	1.56 (1.19, 2.04)	**0.001**
***P* for trend**			**< 0.0001**		**< 0.0001**		**0.004**

HR, hazard ratio; 95% CI, 95% confidence interval; UHR, uric acid to high-density lipoprotein cholesterol ratio. Significance differences were showed as P < 0.05 marked in bold.

Model 1: adjusted for none;

Model 2: adjusted for age and gender;

Model 3: adjusted for age, gender, race, marital status, educational level, family income-poverty ratio, body mass index, smoking status, alcohol intake, cardiovascular disease, chronic kidney disease, hypertension, hyperlipidemia, cancer, and HbA1c.

### 3.3 Non-linear relationships

**Figure 3:**
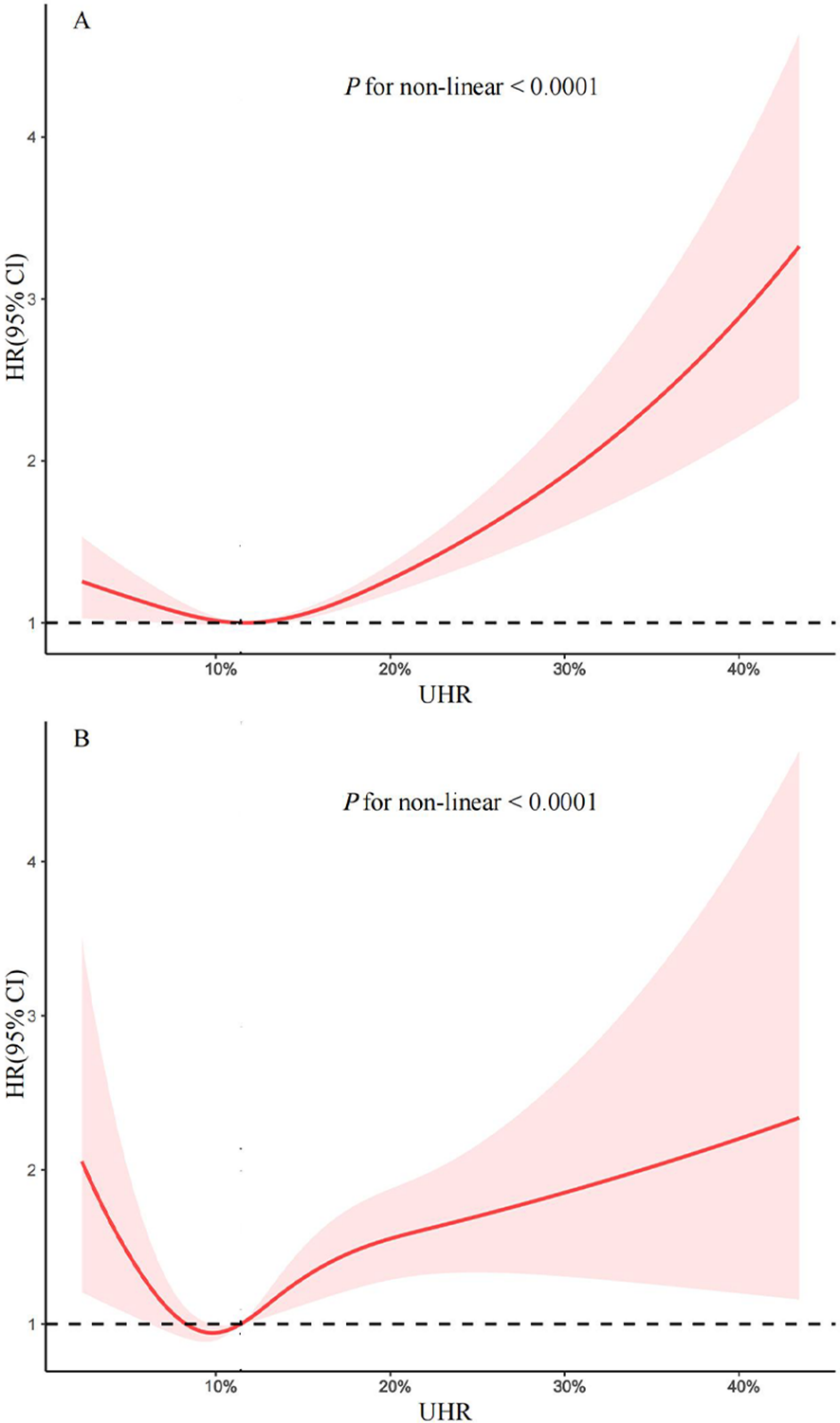
Restricted cubic spline for the association between UHR and all-cause **(A)** and cardiovascular mortality **(B)** in patients with diabetes and prediabetes. Adjusted for age, gender, race, marital status, educational level, family income-poverty ratio, body mass index, smoking status, alcohol intake, cardiovascular disease, chronic kidney disease, hypertension, hyperlipidemia, cancer, and HbA1c. The solid line and red area represent the hazard ratio and their corresponding 95% confidence interval, respectively. UHR: uric acid to high-density lipoprotein cholesterol ratio.

To further investigate the association between UHR and mortality, the adjusted RCS curve was used to illustrate the dose-response relationship of UHR with all-cause mortality and cardiovascular mortality (**Figure 3**). Following comprehensive adjustments for demographics, lifestyle, comorbidities, and glycemic control, a J-shaped association was observed between UHR and all-cause and cardiovascular mortality (both *P* for non-linear < 0.0001). The two-piecewise Cox proportional hazards regression analyses (**Table 3**) indicated that the threshold points for all-cause mortality and cardiovascular mortality were 13.73% (*P* for log-likelihood ratio < 0.001) and 9.39% (*P* for log-likelihood ratio = 0.016), respectively. Notably, when UHR ≥ 13.73%, every 10% increase in UHR was associated with a 45% increase risk of all-cause mortality (HR: 1.45, 95% CI: 1.31-1.61, *P* < 0.0001). When UHR ≥ 9.39%, every 10% increase in UHR was associated with a 38% increase risk of cardiovascular mortality (HR: 1.38, 95% CI: 1.20-1.60, *P* < 0.0001). However, when UHR was below the threshold, there was not a significant association (*P* > 0.05).

**Table 3:** Threshold effect analysis of UHR on all-cause and cardiovascular mortality in patients with diabetes or prediabetes.

	HR (95% CI)	*P* value
All-cause mortality (per 10% increment)		
Fitting by the standard Cox proportional risk model	1.23 (1.14, 1.32)	**< 0.0001**
Fitting by the two-piecewise Cox proportional risk model		
Inflection point	13.73%	
UHR < 13.73%	0.88 (0.75, 1.04)	0.14
UHR ≥ 13.73%	1.45 (1.31, 1.61)	**< 0.0001**
*P* for Log-likelihood ratio	**< 0.001**	
Cardiovascular mortality (per 10% increment)		
Fitting by the standard Cox proportional risk model	1.27 (1.12, 1.45)	**0.0003**
Fitting by the two-piecewise Cox proportional risk model		
Inflection point	9.39%	
UHR < 9.39%	0.59 (0.32, 1.10)	0.10
UHR ≥ 9.39%	1.38 (1.20, 1.60)	**< 0.0001**
*P* for Log-likelihood ratio	**0.016**	

HR, hazard ratio; 95% CI, 95% confidence interval; UHR, uric acid to high-density lipoprotein cholesterol ratio. Significance differences were showed as P < 0.05 marked in bold.

Adjusted for age, gender, race, marital status, educational level, family income-poverty ratio, body mass index, smoking status, alcohol intake, cardiovascular disease, chronic kidney disease, hypertension, hyperlipidemia, cancer, and HbA1c.

### 3.4 Subgroup and sensitivity analyses

With lower UHR (all-cause mortality: < 13.73%, cardiovascular mortality: < 9.39%) as the reference group, the correlation between higher UHR (all-cause mortality: ≥ 13.73%, cardiovascular mortality: ≥ 9.39%) and mortality was analyzed in different subgroups (**Tables 4**, **5**) according to age (< 60 years, ≥ 60 years), sex (male, female), BMI (< 18.5 kg/m^2^, 18.5-25 kg/m^2^, 25-30 kg/m^2^, ≥ 30 kg/m^2^), cardiovascular disease (yes, no), chronic kidney disease (yes, no), hypertension (yes, no), hyperlipidemia (yes, no), and diabetic condition (prediabetes, diabetes). There was no significant interaction effect between UHR and most stratified variables. However, the correlation of UHR with all-cause mortality was significantly modified by sex (*P* for interaction = 0.007) and age (*P* for interaction = 0.006), with a persistent positive correlation observed in women and those aged < 60. Specifically, each 10% increase in UHR was associated with a 31% increase in the risk of all-cause mortality in women (HR: 1.31, 95% CI: 1.13-1.53, *P* < 0.001) and a 35% increase in the risk of all-cause mortality among patients aged < 60 (HR: 1.35, 95% CI: 1.02-1.80, *P* = 0.036). In sensitivity analyses, weighted multivariable Cox regression analyses were performed excluding patients who died within two years of follow-up (n = 543) (**Supplementary Table 1**) or adjusting additionally for antidiabetic, lipid-lowering, and uric acid-lowering medications based on Model 3 (**Supplementary Table 2**), and the results were similar.

**Table 4:** Subgroup analyses of the association between the UHR and all-cause mortality.

All-cause mortality (per 10% increment)				
	HR (95% CI)			
Subgroups	UHR < 13.73%	UHR ≥ 13.73%	*P* value	*P* for interaction
Sex				0.007
Female	Reference	1.31 (1.13, 1.53)	< 0.001	
Male	Reference	1.01 (0.87, 1.17)	0.925	
Age, years				0.006
< 60	Reference	1.35 (1.02, 1.80)	0.036	
≥ 60	Reference	1.10 (0.98, 1.23)	0.121	
BMI, kg/m^2^				0.625
< 18.5	Reference	1.03 (0.07, 14.70)	0.983	
18.5-25	Reference	1.05 (0.82, 1.34)	0.692	
25-30	Reference	1.20 (0.97, 1.49)	0.093	
≥ 30	Reference	1.15 (0.98, 1.35)	0.094	
Cardiovascular disease				0.241
No	Reference	1.14 (0.98, 1.31)	0.081	
Yes	Reference	1.12 (0.94, 1.33)	0.199	
Chronic kidney disease				0.061
No	Reference	1.01 (0.84, 1.21)	0.925	
Yes	Reference	1.25 (1.10, 1.43)	< 0.001	
Hypertension				0.971
No	Reference	1.09 (0.86, 1.38)	0.474	
Yes	Reference	1.15 (1.00, 1.32)	0.050	
Hyperlipidemia				0.118
No	Reference	1.40 (1.02, 1.93)	0.037	
Yes	Reference	1.10 (0.98, 1.24)	0.114	
Diabetes condition				0.298
Prediabetes	Reference	0.98 (0.82, 1.18)	0.850	
Diabetes	Reference	1.26 (1.07, 1.48)	0.005	

HR, hazard ratio; 95% CI, 95% confidence interval; UHR, uric acid to high-density lipoprotein cholesterol ratio; BMI, body mass index.

HRs were estimated using a two-piecewise Cox proportional risk model on both sides of the inflection point. The model used in the subgroup analysis consisted of all covariates used in model 3 except for the variables used for stratification.

**Table 5:** Subgroup analyses of the association between the UHR and cardiovascular mortality.

Cardiovascular mortality (per 10% increment)				
	HR (95% CI)			
Subgroups	UHR < 9.39%	UHR ≥ 9.39%	*P* value	*P* for interaction
Sex				0.137
Female	Reference	1.13 (0.86, 1.47)	0.382	
Male	Reference	0.77 (0.54, 1.10)	0.153	
Age, years				0.410
< 60	Reference	1.24 (0.42, 3.68)	0.701	
≥ 60	Reference	0.98 (0.81, 1.19)	0.854	
BMI, kg/m^2^				0.505
< 18.5	Reference	0.35 (0.09, 1.41)	0.140	
18.5-25	Reference	1.32 (0.87, 1.99)	0.189	
25-30	Reference	0.81 (0.57, 1.15)	0.234	
≥ 30	Reference	1.07 (0.68, 1.69)	0.756	
Cardiovascular disease				0.732
No	Reference	0.87 (0.63, 1.20)	0.395	
Yes	Reference	1.09 (0.82, 1.45)	0.549	
Chronic kidney disease				0.399
No	Reference	0.78 (0.52, 1.18)	0.245	
Yes	Reference	1.16 (0.89, 1.50)	0.283	
Hypertension				0.491
No	Reference	0.72 (0.45, 1.14)	0.158	
Yes	Reference	1.07 (0.83, 1.37)	0.615	
Hyperlipidemia				0.236
No	Reference	0.88 (0.53, 1.44)	0.605	
Yes	Reference	1.01 (0.78, 1.29)	0.966	
Diabetes condition				0.91
Prediabetes	Reference	0.78 (0.56, 1.09)	0.146	
Diabetes	Reference	1.13 (0.84, 1.51)	0.421	

HR, hazard ratio; 95% CI, 95% confidence interval; UHR, uric acid to high-density lipoprotein cholesterol ratio; BMI, body mass index.

HRs were estimated using a two-piecewise Cox proportional risk model on both sides of the inflection point. The model used in the subgroup analysis consisted of all the covariates used in model 3 except for the variables used for stratification.

## 4 Discussion

In this large prospective cohort study of American adults with diabetes or prediabetes, higher UHR was linked to an increased risk of all-cause and cardiovascular mortality. Importantly, these correlations remained significant even after accounting for various potential influencing factors such as demographics, lifestyle, comorbidities, glucose control status, and medication use. In addition, the study revealed a J-shaped association of UHR with the risk of all-cause and cardiovascular mortality, identifying the threshold points (all-cause mortality: 13.73%; cardiovascular mortality: 9.39%). Notably, a higher UHR (≥ threshold) was linked to elevated all-cause mortality in women and those aged < 60 compared to those with a lower UHR. The study underscored the benefits of appropriate management and intervention strategies for serum UA and HDL-C levels in patients with diabetes or prediabetes and the potential of UHR as a prognostic marker.

UA is a metabolite of purines, and hyperuricemia is linked to various adverse health outcomes like chronic kidney disease, cardiovascular disease, and metabolic syndrome (27). As an essential component of lipid metabolism, HDL-C benefits the cardiovascular system by having anti-inflammatory, antioxidant, and vasodilatory properties (28). Previous studies have shown that individuals with elevated serum UA levels and low HDL-C levels have a higher risk of all-cause and cardiovascular mortality in the general population (29, 30). However, this association seems to be more complex in individuals with abnormal glucose metabolism. Several studies examined the potential prognostic value of serum UA in T2DM, but the results have been inconsistent (31–33). Furthermore, a real-world cohort study found no statistically significant connection between HDL-C levels and all-cause and cardiovascular death in individuals with T2DM (34). Nevertheless, a prospective cohort study from the UK Biobank reported that this association may vary by lipoprotein particle size (35). These suggested that a single serum UA or HDL-C levels may not be a good predictor of prognosis for patients with T2DM. UHR, a recently proposed index, combines the cardiovascular risk factor (serum UA) and the cardioprotective factor (HDL-C), associated with inflammation and metabolic disorders. Recent studies have evaluated the clinical significance of UHR in patients with T2DM. Kocak et al. first proposed the UHR and found that it strongly predicted metabolic syndrome and poor glycemic control in T2DM (12). Kosekli et al. reported a significant increase in UHR in individuals with new-onset T2DM (36). Consistent with previous findings (12, 36), we discovered that higher UHR quartiles in diabetes or prediabetes were associated with higher HbA1c levels. Yan et al. explored the relationship between UHR and complications of T2DM in men and postmenopausal women, finding that UHR was positively related to cardiovascular disease and nephropathy but not to retinopathy (37). It is worth noting that individuals with elevated UHR showed a higher prevalence of hyperlipidemia, hypertension, cardiovascular disease, and chronic kidney disease in the current study. These conditions tend to be linked to an increased risk of death, indicating that higher UHR may be associated with poor prognosis. However, it remains unclear whether UHR is linked to elevated mortality in individuals with diabetes or prediabetes.

To our knowledge, this may be the first study to explore the association of UHR with mortality in individuals with diabetes or prediabetes. Our study included 18,804 American adults with diabetes or prediabetes, with a median follow-up of 80 months, revealing that elevated UHR was associated with an increased risk of all-cause and cardiovascular mortality. Limited previous research has explored the prognostic value of UHR (22, 38). Yu et al. reported that high UHR was associated with elevated occurrence of major adverse cardiovascular events (MACEs) and all-cause mortality in patients with acute myocardial infarction, with UHR predicting MACEs and all-cause mortality events with area under the curves (AUC) of 0.716 and 0.711, respectively (22). Furthermore, the study by Yu et al. identified an enhanced prognostic prediction through the interaction between serum UA and HDL-C. Of note, higher serum UA levels and abnormal lipid metabolism (including lower HDL-C levels) are common in individuals with abnormal glucose metabolism (24, 25). The mean (± SE) of UHR in individuals with diabetes or prediabetes was 12.31 ± 0.07%, higher than 10.09 ± 4.23% reported in previous studies in the general population (17). Liu et al. also reported a similar positive correlation between UHR and the risk of cardiovascular deaths in dialysis patients, with subgroup analyses indicating a more pronounced correlation observed in patients with comorbid diabetes (38). Notably, the two studies above had relatively small sample sizes and were limited to the Chinese population. Further exploration and validation are required to determine the prognostic value of UHR.

We also found a J-shaped association between UHR and all-cause and cardiovascular mortality in patients with diabetes or prediabetes. Previous research has also shown a non-linear correlation between serum UA or HDL levels and poor prognosis (39, 40). Lamacchia et al. observed a J-shaped association between serum UA and all-cause mortality in three Italian T2DM cohorts (39). Similarly, Mazza et al. found a J-shaped association between serum UA and coronary mortality in older adults with T2DM (41). Extensive cohort studies from both the United States and China have also indicated that both low and high HDL-C levels are associated with a greater risk of all-cause and cardiovascular mortality (42, 43). Yi et al. identified a U-shaped association between HDL-C and mortality in middle-aged and elderly Koreans (40). The above-mentioned studies may help explain the non-linear relationship between UHR and mortality. In addition, our subgroup analyses revealed a remarkable positive correlation between UHR and all-cause mortality in women and those aged < 60, underscoring the importance of strengthening serum UA and lipid management in these populations to prevent premature death in individuals with abnormal glucose metabolism. Meanwhile, the positive relationship between UHR and the risk of cardiovascular death was not affected by age, sex, various comorbidities, or diabetes conditions. In summary, our findings support the potential prognostic value of UHR in a population with abnormal glucose metabolism, and monitoring and controlling UHR may be beneficial in improving survival.

The association of elevated UHR with increased mortality risk in individuals with abnormal glucose metabolism can be explained by various mechanisms. Overall, it is primarily linked to high serum UA levels, low HDL-C levels, or both. First, serum UA promotes atherosclerosis by inducing vascular endothelial dysfunction, production of inflammatory factors, and by inhibiting autophagy (44, 45). Serum UA can also activate the renin-angiotensin-aldosterone system (RAAS), leading to increased cardiovascular and renal complications (46, 47). Of note, Long-term hyperglycemia exacerbates inflammation, oxidative stress, and activation of the RAAS, ultimately resulting in a poor prognosis for individuals with abnormal glucose metabolism (47). Second, multiple functions of HDL-C are impaired in T2DM, including decreased antioxidant capacity, reduced ability to inhibit inflammatory pathways, and diminished protection of vascular endothelial, all of which indirectly promote the occurrence and development of atherosclerosis (48, 49). Finally, the interaction between high serum UA and low HDL-C may increase inflammation and oxidative stress. On the one hand, it promotes the progression of atherosclerosis and thereby increases the risk of cardiovascular disease (50). On the other hand, it synergistically has adverse effects on cardiovascular, renal, and other organs through IR, thereby increasing the risk of death (51, 52). More importantly, the abnormal state of glucose metabolism exacerbated the link between IR and the risk of cardiovascular disease (53).

There are several limitations of this study. First, Calculating UHR solely based on baseline measurements of serum UA and HDL-C may not capture the dynamic changes over long-term follow-up, potentially impacting the relationship with mortality. Second, despite adjusting for various factors such as demographics, lifestyle, comorbidities, glucose control status, and medication use, residual confounders such as dietary habits and duration of diabetes were not considered. Third, participants in the analysis were exclusively from the United States, and whether the findings are generalizable to other regions remains unknown. Fourth, due to the nature of observational studies, the causal relationship between UHR and mortality may not be established. Despite that, some strengths of our study should be highlighted. First of all, this cohort study utilized an extensive database from a national source with a sizeable sample, a long follow-up period, and relatively reliable data obtained. Second, we used a weighted design according to the NHANES guidelines and performed sensitivity analyses with results consistent with the main findings, thus increasing the credibility of the present study. Finally, we explored the prognostic value of UHR for the first time in diabetic and prediabetic individuals. We also elucidated the threshold points for UHR in all-cause and cardiovascular mortality, respectively. All in all, our study highlights the promising application of UHR in the clinical management of abnormal glucose metabolism populations.

## 5 Conclusions

The study revealed that elevated UHR was linked to a notable rise in both all-cause mortality (threshold point of 13.73%) and cardiovascular mortality (threshold point of 9.39%) in patients with diabetes or prediabetes. These findings emphasize the importance of monitoring UHR as a potential indicator of increased mortality risk. Therefore, proper management and intervention strategies targeted at regulating serum UA and HDL-C levels would be essential in reducing the likelihood of adverse health outcomes in individuals with abnormal glucose metabolism. Further research is necessary to evaluate the prognostic value of UHR as a clinical indicator and to investigate potential underlying mechanisms.

## Data Availability

Publicly available datasets were analyzed in this study. This data can be found here: https://www.cdc.gov/nchs/nhanes/index.htm.
