# Supplementary material for: Association of serum uric acid to high-density lipoprotein cholesterol ratio with all-cause and cardiovascular mortality in patients with diabetes or prediabetes: a prospective cohort study
Source: Front Endocrinol (Lausanne). 2024 Dec 5;15:1476336. doi: 10.3389/fendo.2024.1476336 (PMC11655219; doi:10.3389/fendo.2024.1476336)
Supplement: Supplementary file 1 [file Table1.docx]

Supplementary Material

# Supplementary Tables

**Supplementary Table 1** Sensitivity analysis of UHR on all-cause and cardiovascular mortality in patients with prediabetes or diabetes by excluding participants who died within 2 years.

| **UHR**  **quantiles** |  | **Model 1** | | **Model 2** | | **Model 3** | |
| --- | --- | --- | --- | --- | --- | --- | --- |
|  | **Number of deaths** | **HR (95% CI)** | ***P*** **value** | **HR (95% CI)** | ***P* value** | **HR (95% CI)** | ***P* value** |
| **All-cause mortality** | | | | | | | |
| **Q1** | 511 | 1.07 (0.94, 1.21) | 0.320 | 1.00 (0.87, 1.15) | 0.974 | 1.08 (0.93, 1.25) | 0.299 |
| **Q2** | 496 | Reference |  | Reference |  | Reference |  |
| **Q3** | 525 | 0.97 (0.85, 1.12) | 0.676 | 0.99 (0.85, 1.15) | 0.904 | 0.93 (0.79, 1.10) | 0.409 |
| **Q4** | 673 | 1.35 (1.16, 1.56) | **< 0.0001** | 1.50 (1.28, 1.76) | **< 0.0001** | 1.25 (1.06, 1.48) | **0.007** |
| ***P* for trend** |  |  | **< 0.001** |  | **< 0.0001** |  | **0.044** |
| **Cardiovascular mortality** | | | | | | | |
| **Q1** | 149 | 1.13 (0.90, 1.42) | 0.303 | 1.03 (0.81, 1.31) | 0.816 | 1.24 (0.93, 1.64) | 0.141 |
| **Q2** | 142 | Reference |  | Reference |  | Reference |  |
| **Q3** | 168 | 1.06 (0.82, 1.38) | 0.662 | 1.10 (0.83, 1.45) | 0.521 | 0.99 (0.74, 1.32) | 0.935 |
| **Q4** | 234 | 1.85 (1.45, 2.37) | **< 0.0001** | 2.11 (1.61, 2.76) | **< 0.0001** | 1.65 (1.24, 2.19) | **< 0.001** |
| ***P* for trend** |  |  | **< 0.0001** |  | **< 0.0001** |  | **0.013** |

HR: hazard ratio, 95 % CI: 95% confidence interval, UHR: uric acid to high-density lipoprotein cholesterol ratio.

Model 1: adjusted for none;

Model 2: adjusted for age and gender;

Model 3: adjusted for age, gender, race, marital status, educational level, family income-poverty ratio, body mass index, smoking status, alcohol intake, cardiovascular disease, chronic kidney disease, hypertension, hyperlipidemia, cancer, and HbA1c.

**Supplementary Table 2** Sensitivity analysis of UHR on all-cause and cardiovascular mortality in patients with prediabetes or diabetes by further adjusting for drugs based on model 3.

| **UHR**  **quantiles** |  | **Model 4** | |
| --- | --- | --- | --- |
|  | **Number of deaths** | **HR (95% CI)** | ***P*** **value** |
| **Q1** | 620 | 1.01 (0.88, 1.16) | 0.861 |
| **Q2** | 621 | Reference |  |
| **Q3** | 653 | 0.91 (0.78, 1.05) | 0.188 |
| **Q4** | 854 | 1.24 (1.07, 1.45)) | **0.006** |
| ***P* for trend** |  | **0.006** |  |
| **Q1** | 181 | 1.17 (0.91, 1.51) | 0.220 |
| **Q2** | 172 | Reference |  |
| **Q3** | 216 | 1.00 (0.76, 1.31) | 0.993 |
| **Q4** | 300 | 1.57 (1.20, 2.06) | **< 0.001** |
| ***P* for trend** |  | **0.005** |  |

HR: hazard ratio, 95 % CI: 95% confidence interval, UHR: uric acid to high-density lipoprotein cholesterol ratio.

Adjusted for age, gender, race, marital status, educational level, family income-poverty ratio, body mass index, smoking status, alcohol intake, cardiovascular disease, chronic kidney disease, hypertension, hyperlipidemia, cancer, HbA1c, **antidiabetic drugs, antilipemic drugs, and urate-lowering drugs.**
